# Uplifting of anticorrosive coatings performance via TiO_2_/ZnO core–shell pigment for oil and gas pipelines protection

**DOI:** 10.1038/s41598-023-47417-w

**Published:** 2023-11-17

**Authors:** N. M. Ahmed, M. G. Mohamed

**Affiliations:** Polymers and Pigments Department, National Research Centre, Dokki, Cairo, Egypt

**Keywords:** Chemical engineering, Chemistry publishing

## Abstract

The objective of this study is to enhance the effectiveness of anticorrosive coatings applied to steel pipelines utilized for the transportation of petroleum products. However, these pipelines are susceptible to corrosion, necessitating the implementation of an economically viable protection system. Therefore, this research endeavors to introduce a novel pigment consisting of titanium dioxide/zinc oxide (TiO_2_/ZnO) core–shell structure. The pigment is designed to be incorporated into polymeric coating formulations and subsequently subjected to standard testing methods, including immersion tests, adhesion assessments, and electrochemical measurements. The pigment was synthesized and characterized using X-ray diffraction (XRD), X-ray fluorescence (XRF), and electron microscopy (SEM and TEM). Electrochemical impedance spectroscopy (EIS) was hired for observing the resistance and capacitance of the formulated coatings through immersion in corrosive medium (3.5% NaCl). The obtained results conveyed that the prepared core–shell pigment with its unique structure had strongly elevated the resistivity of the formulated coating, which enhanced its role in the protection of the pipeline wall from corrosion.

## Introduction

Corrosion is a striking industrial setback. Its improvement and the substitution of the affected parts by others are high cost accomplishments. In oil and gas industry, corrosion is encountered in the down-hole and the surface equipment of the oilfield, including wells, pipelines, tanks, heat exchangers, and separators^1^. Crude oil is a very corrosive fluid that contains organic acids, salt water, and dissolved gases, such as oxygen (O_2_), carbon dioxide (CO_2_), and hydrogen sulfide (H_2_S). This composition elevates the conductivity and enhances corrosion. In addition the presence of CO_2_ and H_2_S has the propensity to be deionized, thus decreasing the pH of the fluid which can be a further of additional corrosion.

In general, corrosion of pipelines can be classified mainly to external and/or internal corrosion. External corrosion takes place according to the surrounding environmental effects on the pipelines metallic parts, which is often associated with high temperature, salt, humidity and acidic environments. Whereas, internal corrosion is directly associated with the type of transported materials; which is basically caused by the continuous exposure to the transported fluid^2^.

Pipelines are habitually made of low-carbon steel. In the petroleum industry, these pipelines are prone to corrosion, which obliges regular alternating substitutions. To lessen the substitution costs, corrosion-resistant alloys can be exploited on behalf of carbon steel. However, these alloys are much pricey than low-carbon steel; and hence their use is limited to special parts. One way to diminish corrosion is the use barrier coatings, to protect the outer surface of the pipeline from corrosive materials and conditions^3^. Demands for corrosion-resistant coatings include good adhesion to low-carbon steel but low surface energy (to prevent deposition on the steel surface). It also needs resistance to high operational temperatures and pressures, resistance to corrosive chemicals, flexibility, ease of application, and low cost. Various materials have been suggested and studied for this purpose^4^.

Diamond-like-carbon coatings possess high wear and corrosion resistance, and they have revealed good barrier and anticorrosion properties^5^. Ni–P/SiC composite coatings also demonstrated good wear and corrosion resistance^6,7^. However, both these materials are pricey and require unusual application techniques; e.g. chemical vapor deposition. Therefore, they are not suitable for application or reparation.

High-performance polymeric coatings are premium candidates that can avoid corrosion; they are of low cost and are susceptible to ease of application or reparation. Pigment in any formulation can either improve or corrupt the whole performance of the protective coating. Increasingly, experts are seeking pigments that can correlate the relationship between functionality and performance, to impart superior durability to the finished coating as an end outcome. Appropriate pigmentation not only constrains the aesthetics of the film, such as gloss, opacity and color, but also they enhance many other mechanical and performance properties, as well as the level of transformations that are created by the environment upon such properties^8^.

Core–shell preparation method presents a new course to attain structured-particle pigments to offer high performance, functional anticorrosive pigments with associated savings. This theory is based on depositing a thin surface layer of efficient anticorrosive pigment on a bulk material which is the major content of the pigment^9,10^. The combination of both core and shell compounds can lead to the creation of new pigments with better assets, which is different from each of its entity elements. A good choice of both candidates can produce high performance structured-pigment with enhanced properties that can conquer the deficiencies of its individual components; and consequently they cannot only improve the properties of the new tailored-pigments that guide the change of their role and performance, but also they can prevail over the deficiencies that were initially present in both candidates.

The main track of this work is around the formulation of an organic coating based on epoxy two packs system with the new core–shell pigment (TiO_2_/ZnO) which is composed of titanium dioxide as a shell on core of zinc oxide. As might be predicted from the combination of these two candidates, a production of high performance core–shell pigment can be launched as high performance anticorrosive candidate in organic coatings, and its application will be more fitting as an exterior coating on the surface of the pipelines more than being in the interior.

## Experimental work

### Materials

Zinc nitrate is white powder of purity 99% obtained from Win-lab, UK. Titanium (III) chloride solution is used as source of titanium oxide which obtained from Sigma-Aldrich. Liquid Ammonia Solution (25%) was obtained from Loba chemie lab, Mumbai, India with specific gravity of 0.91. Epoxy resin used is two-component system with Bisphenol A type resin and polyamide hardener used with (1:4 resin: hardener). The used solvent is xylene rectified which obtained from Lobachemi lab, Mumbai, India. Its molecular weight 106.17 and its assay is 98.5%.

### Preparation of TiO_2_/ZnO core–shell pigment

**Figure 1:**
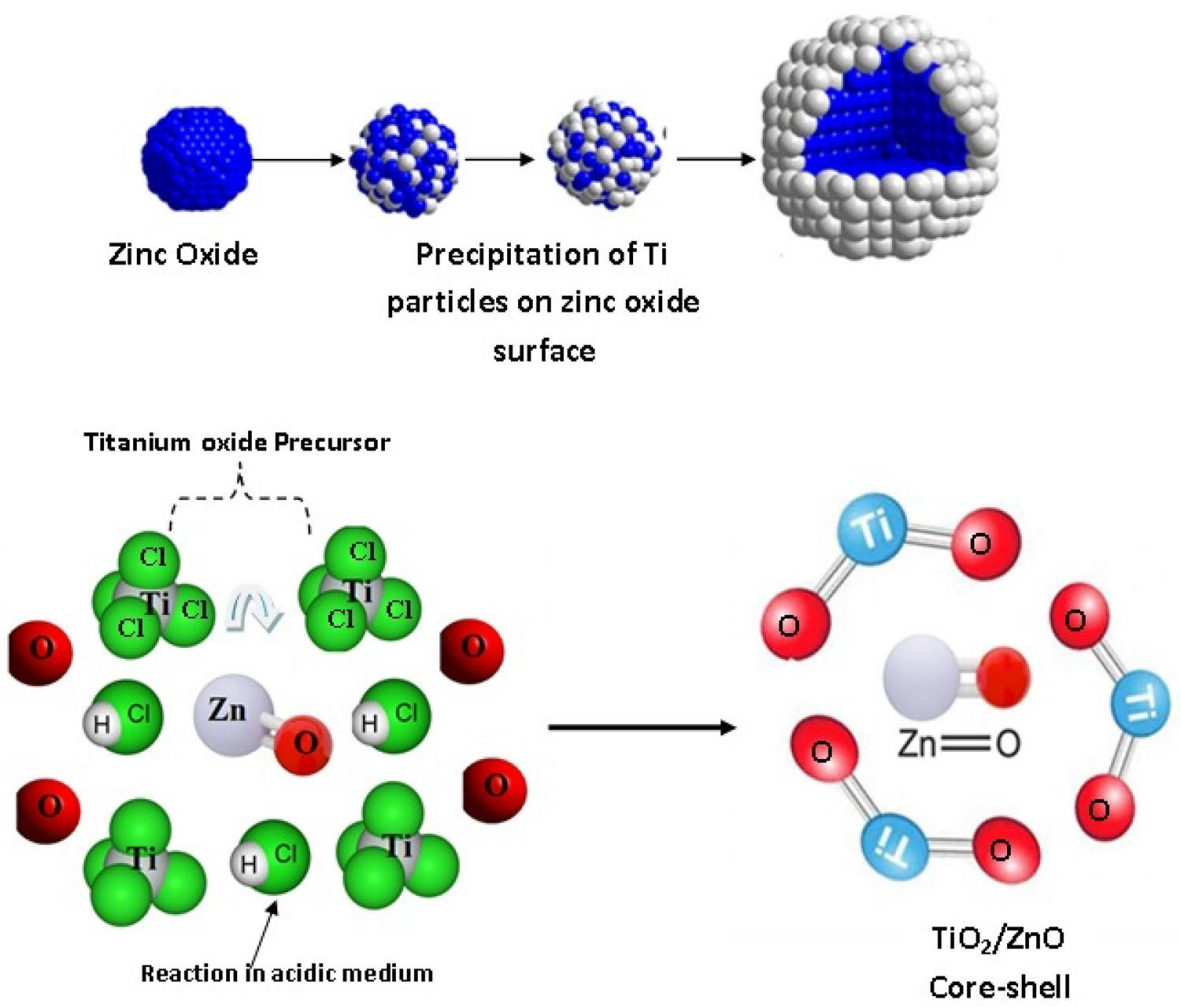
Preparation of TiO_2_/ZnO core–shell pigments.

The preparation was preceded according to the following steps;

Step 1, titanium oxy-hydroxy salt was added to 100 ml hydrochloric acid with vigorous stirring then zinc oxide was immersed in this solution and left for adequate time during slow stirring rate to assure complete covering.
Step 2, the prepared solution in step one was poured in slight acidic medium, then ammonia solution was added drop-wisely to this mixture to set the pH to the neutral phase till complete precipitation of the titanium dioxide layer.
Step 3, filtration through a Buchner system followed by washing the product very well then calcining it at 500–750 °C was done. The calcined powder was then subjected to ball milling for adequate time with high rpm (300/min) to reach the required particle size. A schematic diagram represents the preparation process can be found in Fig. 1.

### Coating preparation

In this part, epoxy resin (two component) which is the resin and its hardener with a ratio of (1:4 resin: hardner) were added in four coating formulations to zinc oxide, titanium dioxide and TiO_2_/ZnO core–shell pigment. Also, talc and iron oxide were added as extenders, and the pigments were loaded with about 25% of the total solid content of the formula as shown in Table 1. The formulations were prepared via high speed ball milling to assure the complete homogeneity of all the ingredients in the coating solution. The coat was then applied on steel using brush to get dry film thickness between 100 and 150 µm.

**Table 1:** Coating formulations.

Ingredients (gm)/coat	Coating containing ZnO	Coating containing TiO_2_	Coating containing TiO_2_/ZnO core–shell
Fe_2_O_3_	7	7	7
Kaolin	12	`12	`12
Talc	10	10	10
ZnO	29	–	–
TiO_2_	–	29	–
TiO_2_/ZnO Core–shell pigment	–	–	29
Total pigment	58		
Epoxy Resin (2K)	42		
Total	100		

### Pigment characterization

#### X-ray fluorescence (XRF)

The concentrations of the different oxides in the prepared pigments were determined using Axios, sequential WD-XRF spectrometer, PANalytical 2005, USA.

#### X-ray diffraction (XRD)

X-ray powder diffraction patterns were obtained at room temperature using a Philips diffractometer (Model PW 1390) Japan, employing Ni-filtered Cu Kα radiation (λ = 1.5404 Å). The diffraction angle (2θ) was scanned at a rate of 2°/min.

#### Transmission electron microscopy (TEM)

The prepared pigment was inspected by means of (JEOL JX 1230) technique with micro-analyzer electron probe (Japan). This technique shows a cross-section of the particles and determines the particle sizes of the prepared pigments.

#### Scanning electron microscopy/Energy dispersive x-ray analysis (SEM/EDX)

Energy-dispersive X-ray analysis technique and scanning electron microscopy (JEOL JX 2840) micro-analyzer electron probe (Japan) was used in this work to estimate the particle shapes and the elements located on the surface of the formed compound.

### Preparation of test samples

In the beginning the oxide/hydroxide, mill scale and other kinds of noxious wastes were removed from the steel substrates. Then, all anticorrosive coating formulations were applied on carbon steel panels of dimensions (100 mm × 120 mm × 0.8 mm) using a brush. The brushing was done to get a smooth, uniform coat thickness as possible without leaving brush marks, runs, or sags. After that, the coatings were stored for 48 h for hardening at temperature 23 ± 2 °C and relative humidity at 40 ± 5%. The dry film thickness of all coating films were then adjusted to be 150–200 µm and were measured using the magnetic thickness gauge.

### Mechanical properties

The mechanical properties which include the impact resistance, ductility and hardness of the dry paint film were tested according to following standards;

- Hardness of the paints using the pendulum apparatus to study the film elasticity (ASTM D4366-16, 2016).
- Impact resistance (ASTM D6905-03, 2012) which reveals the height of the free fall of a weight (1000 g) at which the paint film still resists impairment.
- The resistance of the coating against cupping in Erichsen cupping tester (ASTM D 5638-00, 2013) is to identify the resistance of the paint film against distortion of a coated steel panel with a pressed-in 20 mm steel ball. The result of the test deduces cupping in mm with the first disturbance of the coating.

### Corrosion studies

#### Immersion test

The immersion test was achieved by pertaining X-scribe of 1 mm width to the coatings along the coupons to expose the underlying metal to the aggressive environment. After 28 days of exposing the panels to saturated 3.5%NaCl solution and they were evaluated for;

- Degree of rusting according to (ASTM D610-08, 2012).
- Degree of coating adhesion (wet adhesion) by means of a cross-cut test according to (ASTM D3359-17, 2017).
- Degree of blistering on painted steel surfaces according to (ASTM D714-02, 2017).
- Filiform corrosion resistance test photographic assessment according to (ASTM D2803-09, 2015).

#### Electrochemical measurements

The corrosion protection performance of coatings was explored using EIS (OGFEIS), which is obtained from Origalysis, France. Three electrode system was used; reference electrode which is silver/silver chloride electrode, the auxiliary electrode which is a platinum sheet and the coated steel coupon (2 × 2 cm) representing the working electrode. EIS measurements were carried out by applying 10 mV sinusoidal potential through a frequency domain from 100 kHz to 100 MHz. The EIS measurements were taken at different interval periods during the immersion time (28 days) in 3.5%NaCl electrolyte and to study the corrosion protection efficiency.

## Results and discussion

### Characterization of the prepared core–shell pigment

#### X-ray fluorescence (XRF)

XRF technique is used to detect the different oxides found in the compound. The results in Table 2 indicated that, zinc oxide percentage was about (79.6%) while titanium dioxide percentage was (14.3%).This result conveyed that zinc oxide (core) was found as a major compound comprising about (85%) of the whole compound, while titanium dioxide (shell) was detected in fewer amounts and this is in good agreement with “core–shell theory”. The percentage of titanium dioxide expresses its whole concentration in the bulk and on the surface; therefore another technique was hired to detect the elements located on the surface only (EDX).

**Table 2:** XRF of TiO_2_/ZnO core–shell pigments.

Main constituents (wt. %)	TiO_2_/ZnO core–shell
ZnO	79.6
Fe_2_O_3_	0.26
Al_2_O_3_	0.95
TiO_2_	14.3
SiO_2_	1.93
MnO	0.17
CuO	0.01
MgO	1.23
CaO	0.36
P_2_O_5_	0.01
SO_3_	0.11
Cl	0.004
LOI	1.06

#### Morphology of core–shell pigments

**Figure 2:**
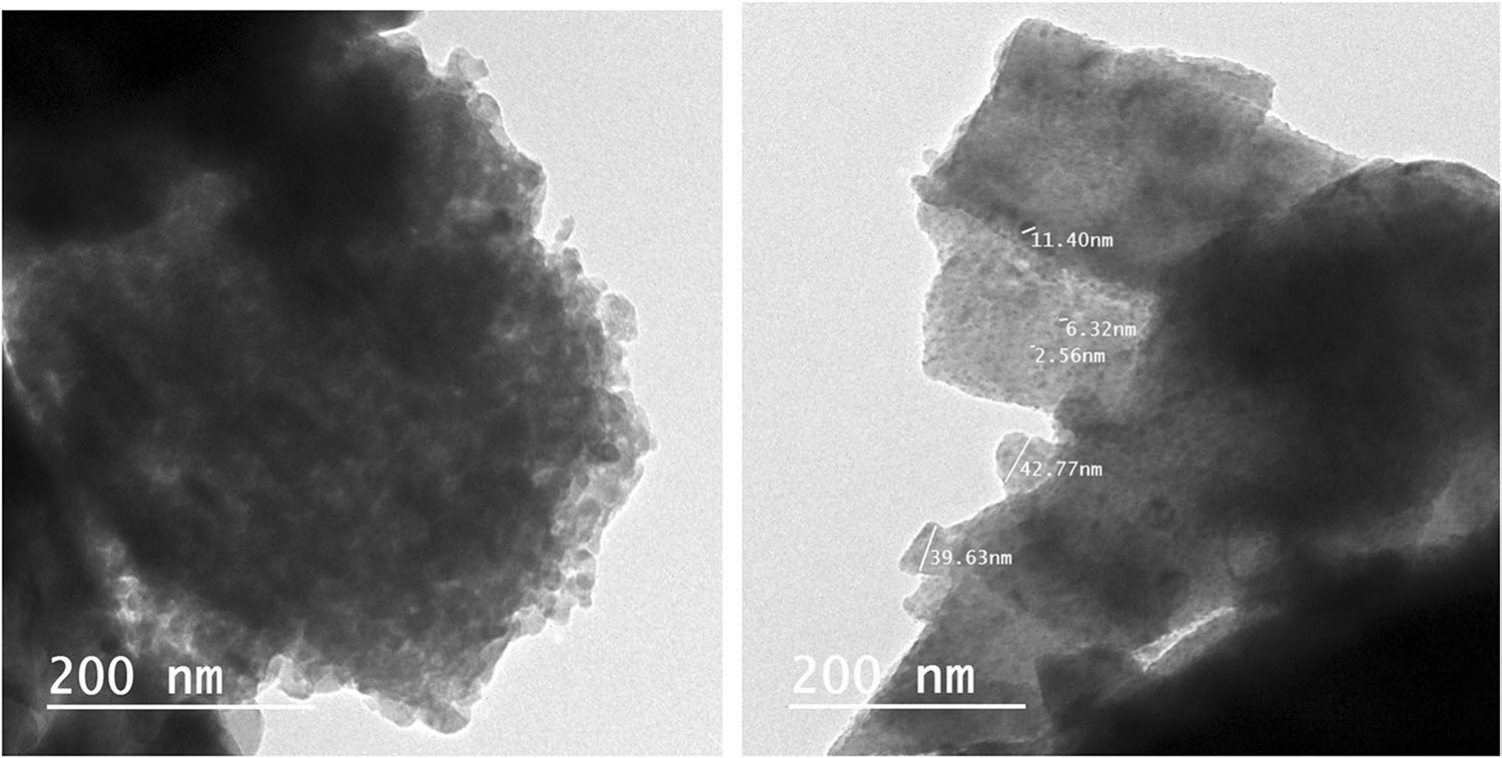
TEM photos of TiO_2_/ZnO core–shell.

**Figure 3:**
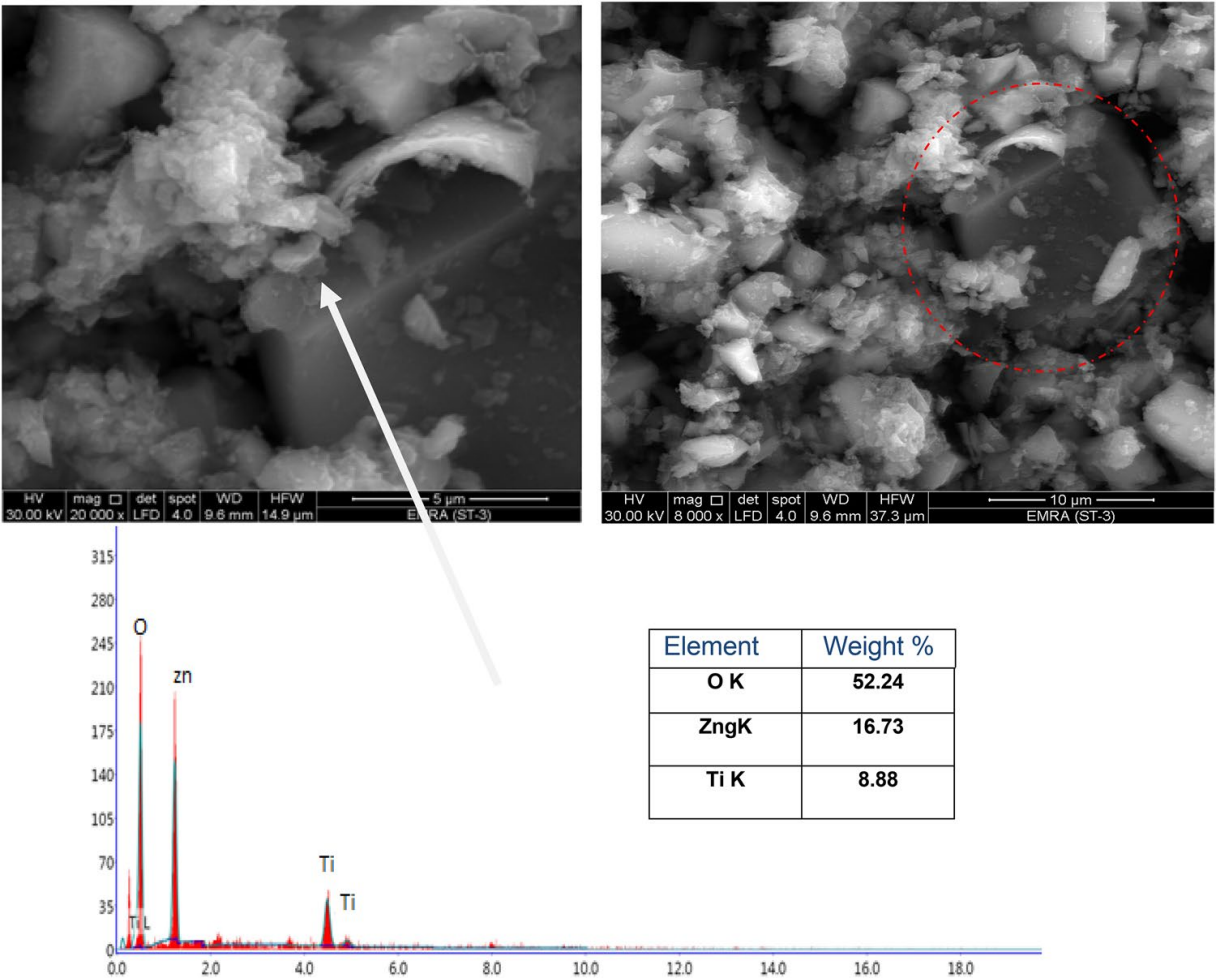
SEM/EDX of TiO_2_/ZnO core–shell.

The particle sizes and the main morphological features introduced by TEM and SEM techniques of the prepared core–shell pigment are shown in Figs. 2, 3. The photos cleared that; zinc oxide was distinguished by its hexagonal large plates that overlap with each other. On the surface of these plates, small spheroid tiny platy stacked particles (titanium dioxide) were allocated and distributed uniformly. This structure was a manifest of the successful preparation of core–shell particles.

EDX analysis which can detect elements up to 1µm depth on the surface was done on the prepared core–shell pigment. The results revealed the presence of zinc oxide as intense peak, while the peak of titanium dioxide was lower in its intensity, which insures that titanium dioxide is present only on the surface as thin layer and not in the bulk, and that it is partially enfolding the core. This means that, the core effect is partially hidden, i.e. both candidates effect is present and they can beneficiate the coat with their characteristics and advantages.

#### X-ray diffraction (XRD)

**Figure 4:**
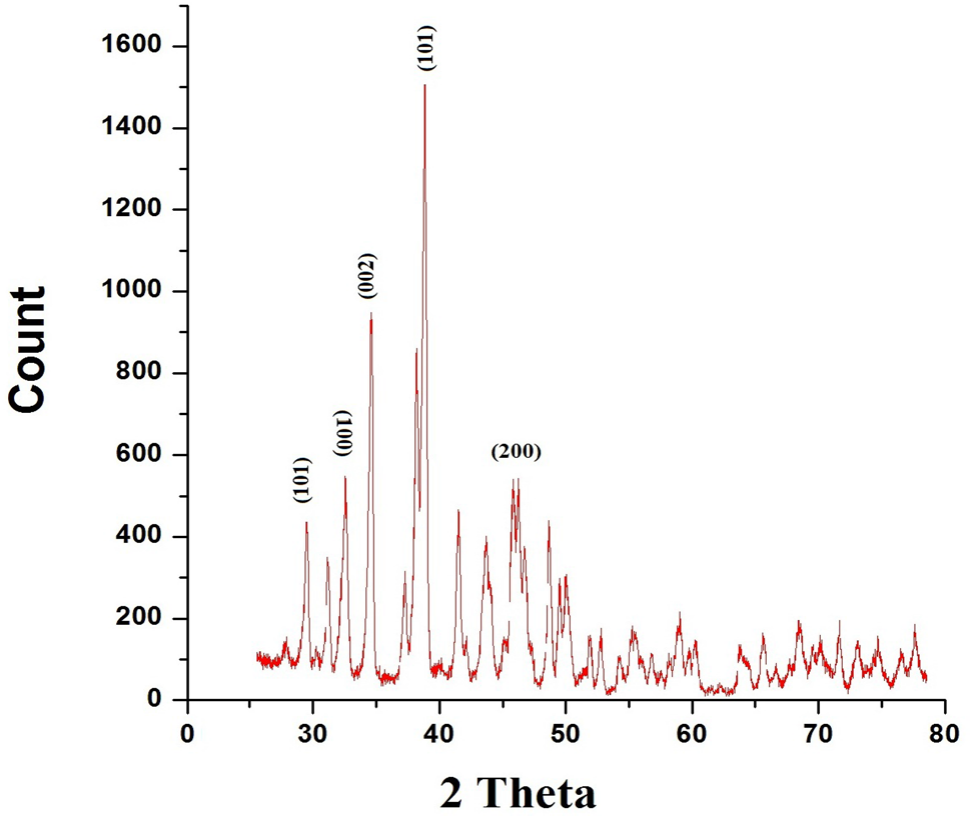
XRD of TiO_2_/ZnO core–shell pigment.

XRD technique is trust worthy to detect the nature of crystallinity of the prepared compound. Figure 4 showed the typical XRD pattern of hexagonal ZnO phase (JCPDS card, PDF card No. 36-451).The peaks are matching with (100), (002) and (101) planes and they are observed in pure ZnO (wurtzite) phase with the hexagonal structure of ZnO in addition to the tetragonal TiO_2_ phase (JCPDS card, PDF card No. 21-1276)^9,10^.

Generally, all the results of XRF, XRD, EDAX, TEM and SEM confirmed the structure of the formed core–shell pigment as grainy thin shell of titanium dioxide covering a bulk of zinc oxide. The intense peaks of zinc oxide in EDX and its concentration that was detected in XRF revealed that the shell is partially shielding the zinc oxide (core), and such structure helps in accompanying zinc oxide in the reaction with the adjacent media, i.e. both components can play their roles in preventing corrosion via their different mechanisms and reactions.

### Mechanical properties of the different coatings

**Figure 5:**
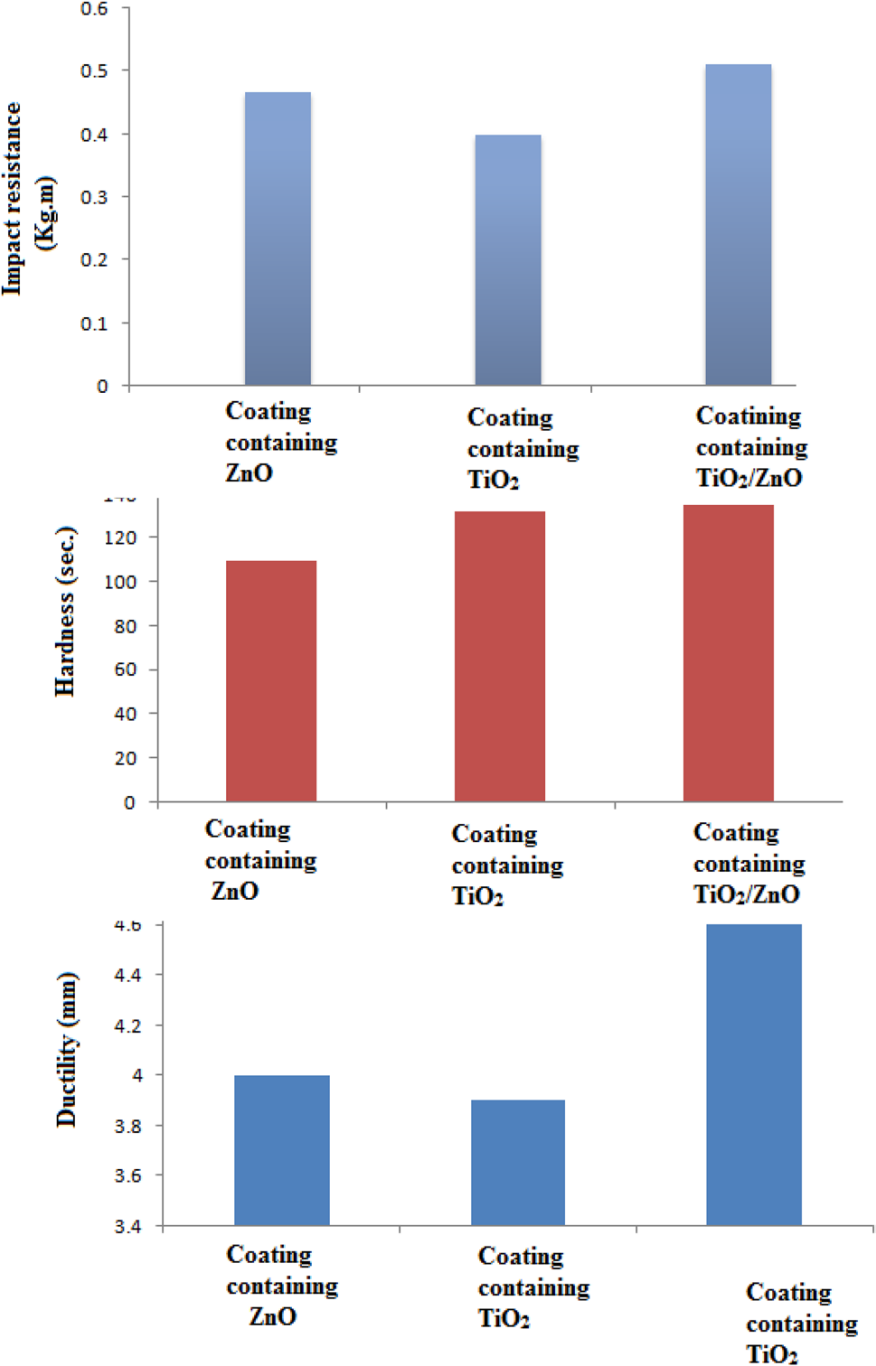
Mechanical properites of different coating formulations.

Only few coating materials combining excellent mechanical strength accompanied with high corrosion resistance can be found; because these properties usually contradict each other^10^. The mechanical properties are important when coatings are applied in harsh environment like that found around the pipelines. The aggressive environment and flow velocity affect directly the coating film and can leave a lot of deformed points through the coating system. Therefore, coatings applied on pipelines must possess good mechanical properties (e.g. ductility, impact resistance and hardness) to outstand efficiently against this aggressive environment both in the outer atmosphere and inside the pipelines. The results of mechanical testing of coating containing zinc oxide, titanium dioxide and TiO_2_/ZnO core–shell pigments are illustrated in Fig. 5. Coating films containing prepared core–shell pigment showed better mechanical properties than those containing the individual zinc and titanium dioxides. The traditional assumption presumed that plate structures of zinc and titanium dioxides enhanced the adhesion, elasticity and reinforced the polymeric matrix of the coating film, therefore titanium and zinc oxides are good candidates to reinforce the epoxy matrix and hence improved mechanical properties can be achieved^11^. Keeping in mind that this can be achieved using individual zinc and titanium dioxides, preparing a composite compound containing various platy structures of different sized overlapping on each other, can exhibit a dual effect and can strongly reinforce the epoxy matrix, i.e. instead of reinforcing the polymeric matrix of the coat with different platy particles, core–shell structured particles of different plate sizes can work as gears that can connect and work with each other to offer the coat its required hardness beside possessing the advantage of being elastic, which makes these coatings withstand efficiently the different impacts^12^.

### Immersion test

**Figure 6:**
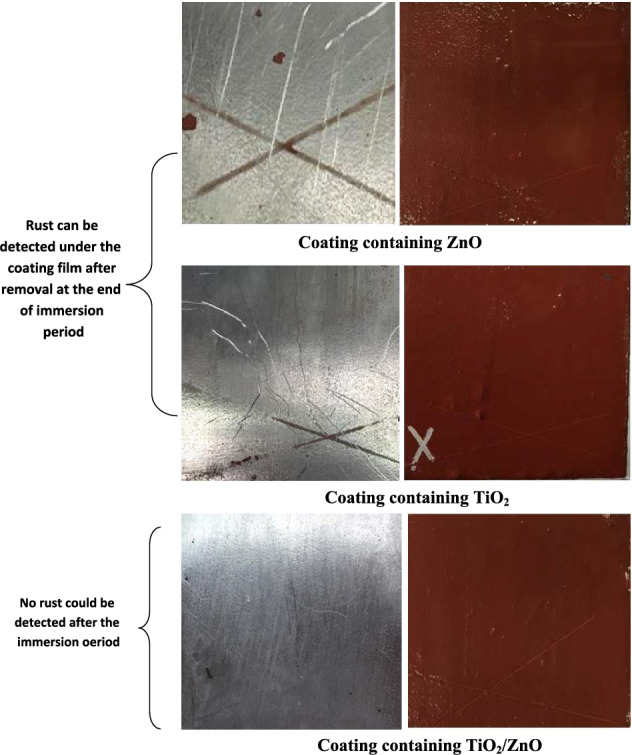
Photo of different coatings after immerion in 3.5%NaCl for 28 days.

Coatings were pertained on steel panel surfaces; hence the accelerated corrosion test was done for 28 days in artificial sea water. After the exposure, the assessment of blistering, adhesion and corrosion protection was executed. The blistering was based mainly on the size and frequency of the formed paint blisters, while the evaluation of the corrosion of etched specimens of both the depth under the film of corroded areas in the etched places and the whole corroded area after coating elimination was assessed. Results of these tests are described in Tables 3 and Fig. 6.

**Table 3:** Immersion test results for different coatings after immersion in 3.5%NaCl for 28 days.

Test	Coating containing ZnO	Coating containing TiO_2_	Coating containing TiO_2_/Zn core–shell
Adhesion	Gt2	Gt3	Gt0
Degree of blistering	10	10	10
Degree of rusting	8-G, 3%	4-G, 10%	10

Note 1: Adhesion can be ordered in descending order as follows; Gt0 > Gt1 > Gt2 > Gt3 > Gt4.

Note 2: Blister density = 10 (i.e. no blisters were observed on the paint films).

Note 3: Degree of rusting = 10 (i.e. no corrosion was observed on the steel films under paint, in the table, degree of rusting is G means (General rusting).

The assessment of blistering degree on the coating surface, conveyed that no blistering were originated on the entire coatings except those containing zinc oxide which showed up blistering of low size and frequency (Table 4).

**Table 4:** Polarization resistance values obtained from EIS measurement.

Rp values (Ohm Cm^2^)			
Time of immersion	Coating containing ZnO	Coating containing TiO_2_	Coating containing TiO_2_/ZnO
1 day	6431	5560	14,301
7 days	8930	3906	22,430
14 days	8740	4101	17,932
21 days	6750	2350	15,001
28 days	7409	2104	14,325

The results of the adhesion after the immersion time were sounded for all the examined coatings, and coated films containing core–shell pigment showed higher adhesion than those containing individual zinc and titanium oxides. It was also obvious from the results of accelerated corrosion test, that the corrosion resistance of coatings containing core–shell pigments (TiO_2_/ZnO) was better than films containing zinc and titanium dioxide individually by exhibiting no rust under the coat while coatings containing zinc and titanium dioxide showed rust under film ranging between (4–10%).

It is also well-known that the inclusion of zinc oxide in the epoxy coatings for prevention of steel improves the corrosion resistance of coated steel by producing some zinc ions which can origin at some products through particular electrochemical reactions that can act as physical barriers especially in dry/wet cycles^13^. While in films including titanium dioxide, TiO_2_inhibits the surface micro-pores, preventing by that the corrosive species from penetrating or diffusing through its good physical barrier^14^. The good corrosion protection of coating containing TiO_2_/ZnO can be explained through the core–shell structured-pigment which can offer dual effect of both electrochemical and physical barriers that are exhibited by the two components (zinc and titanium dioxide).

### Electrochemical impedance spectroscopy (EIS)

**Figure 7:**
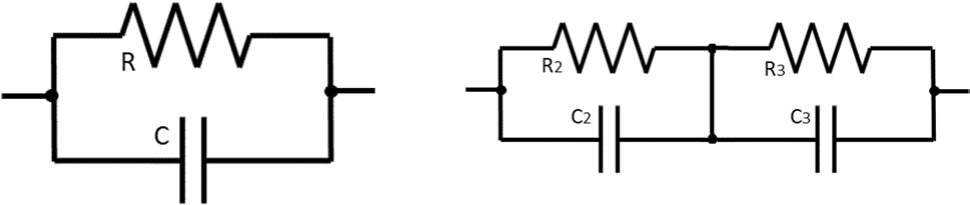
Equivalent circuits.

Electrochemical impedance measurements are commonly used to analyze the behavior of any coating system by fitting the impedance data to an equivalent circuit. This circuit represents the physical processes occurring within the system under investigation. The selected circuits are presented in Fig. 7 which chosen based on different literature and most fitted to the obtained date^15^, typically includes the following elements;

1. **Solution resistance (Rs),** which represents the resistance of the test electrolyte between the working (coated steel) and reference electrodes. It accounts for the conductivity properties of the solution.
2. **Constant phase element (CPE),** which is used instead of a simple capacitor that represents the dielectric properties of the coating. It compensates for any non-homogeneity within the system, providing a more accurate representation of the coating behavior.
3. **Polarization resistance (Rp),** which reflects the corrosion resistivity of the system. It is related to factors such as porosity and corrosion. By analyzing the electrical parameters of the formulated coatings, it is possible to fit the obtained data and extract valuable information about the anticorrosive properties of the coating.

By monitoring the changes of these different parameters over time, particularly during immersion, valuable insights can be gained regarding the performance and the properties of the anticorrosive coating.

**Figure 8:**
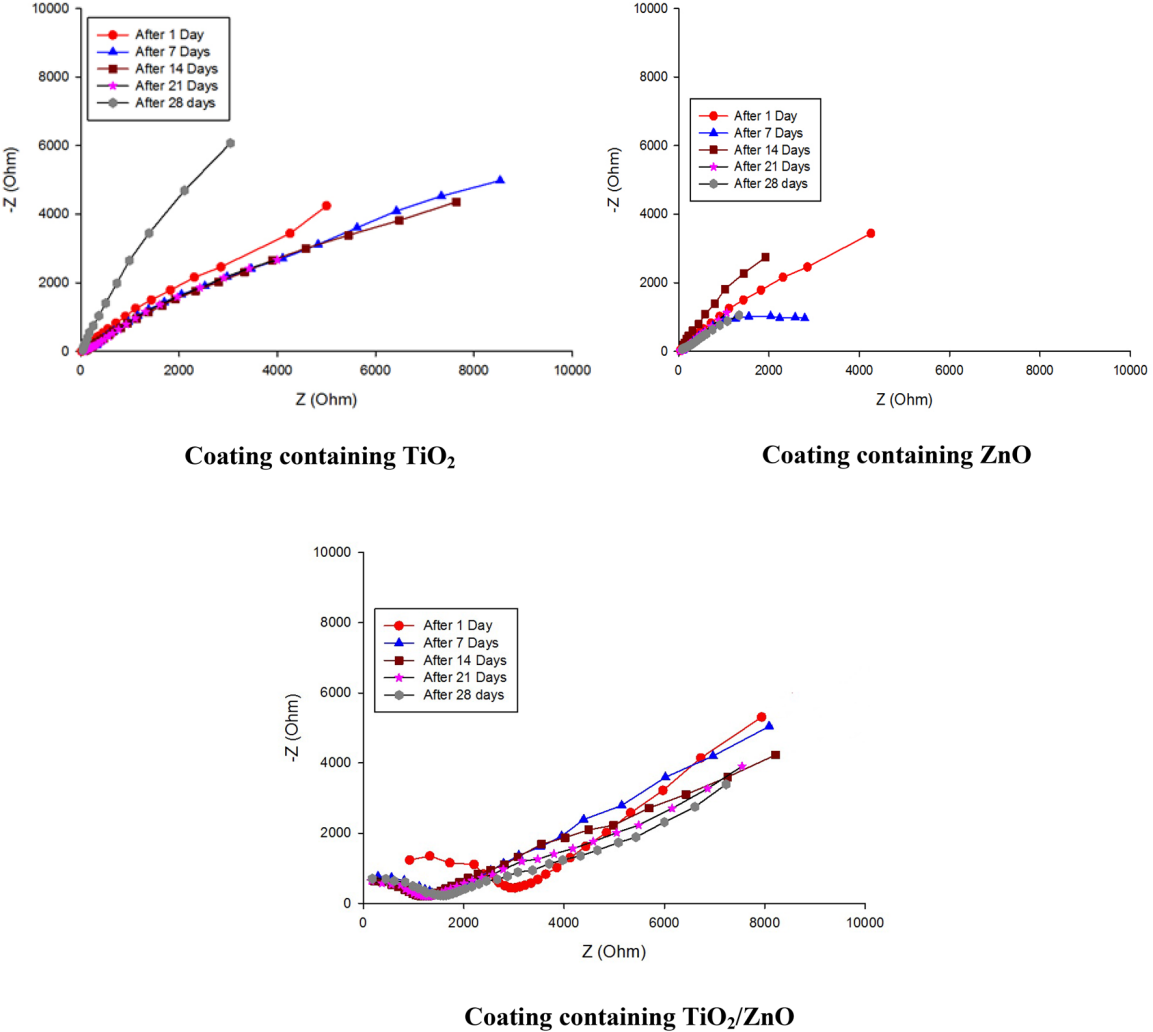
EIS for different coatings during immerion in 3.5%NaCl for 28 days.

Figure 8 displays the Nyquist plots of the formulations incorporating zinc oxide, titanium oxide and core–shell pigment. Coatings containing core–shell pigment displayed two semi-circles in the plot. The first semi-circle was observed in the high-frequency region, and it represents the capacitive reactance of the coating/medium interface. The second semi-circle that was in the low-frequency region corresponds to the metal coating/medium interface within the pores. When the coatings were immersed in 3.5%NaCl solution, changes were observed in the Nyquist diagram during the first week. The bottom of the low-frequency region was decreased in its size due to the presence of pores in the coating. As the corrosive medium penetrated the coating through these pores, electrochemical reactions ensued at the pore/solution interface, resulting in a reduction in the polarization resistance. Prolonged immersion facilitated the infiltration of the medium through micro-pores, intensifying localized corrosion. Impedance measurements are influenced by, the interplay between frequency and various physical and chemical processes occurring within the electrochemical cell and at the corroding interface. Tracking the electrical parameters over the immersion period revealed a decrease in the polarization resistance (Rp) for the blank specimen, reaching 100 Ohms by the end of the immersion period. Coatings containing individual zinc and titanium oxides exhibited some fluctuation in Rp values throughout the immersion period, with high rates of decrease, reaching 834.3 Ohms for the coating containing zinc oxide and 874.2 Ohms for that including titanium oxide. On the contrary, the coating containing the core–shell pigment initially demonstrated a shrink in the polarization resistance during the first two weeks of immersion. However, this shrink reached a stable state by the third week and started to increase once again towards the end of the immersion period.

The potentio-dynamic curves and immersion test results of the various coatings clearly expressed that the coating film containing the prepared core–shell pigment exhibited superior corrosion resistance compared to coatings containing individual zinc and titanium dioxides as showed in previous studies incorporated zinc and titanium oxides as main components for corrosion resistance^16,17^.

The enhanced corrosion resistivity of the coatings with the prepared core–shell pigments can be conveyed to several factors. First, zinc-based pigments are assumed to undergo partial dissolution, releasing selective ions. By incorporating the prepared core–shell pigment with its high zinc content into the anticorrosive coatings, it directly participates in inhibiting the initiation of corrosion reactions through this partial ionization process. The dissolved zinc ions can reach the metal/coating interface and react with OH^−^ ions generated at the cathodic sites on the metal surface, forming protective oxy-hydroxy layers^18–20^.

Moreover titanium dioxide particles in the shell can align themselves on the zinc particles. This alignment provides additional shielding properties to the coating, by blocking the pathways for corrosive materials, and preventing them from penetrating through the coating layers to reach the steel surface. As a result the corrosion reaction is delayed, blistering on the coating film is inhibited, and rust formation under the film is prevented.

In summary, the incorporation of the prepared core–shell pigment in the coatings noteworthy improved corrosion resistance due to the combined effects of the partial ionization of zinc-based pigments and the shielding properties offered by the alignment of titanium dioxide particles on the zinc particles^21,22^.

## Conclusions

The main finals that can be conveyed from the work is that adjoining TiO_2_/ZnO in a core/shell pigment can produce high quality candidate for increasing the durability of epoxy coatings in aggressive exposure conditions. This conclusion is based on the following experimental results:

1. The new core–shell pigments were prepared using simple preparation technique resulting in the formation of new pigment combining two of the most well-known anticorrosive pigments which are TiO_2_ and ZnO in one compound.
2. The different characterization methods have proved the formation of titanium dioxide as thin shell on the surface of zinc oxide without any known chemical bonding which permits to exploit the advantages of both materials.
3. Results obtained from the different tests (immersion test with the visual inspection and EIS) showed that formulation containing core–shell pigments exhibited extreme improvement in the anti-corrosion performance.
4. Also coatings containing the core–shell pigment showed very good mechanical properties and high durability over the other tested coatings which make it a premium candidate to be applied for the defense of oil and gas pipelines.

## Data Availability

All data generated or analysed during this study are included in this published article.

## References

[1] Bayram TC, Orbey N, Adhikari RY, Tuominen M (2015). FP-based formulations as protective coatings in oil/gas pipelines. Progress Org. Coat..

[2] Breton T (2010). Identification of failure type in corroded pipelines: A Bayesian probabilistic approach. J. Hazard. Mater..

[3] Mohamed MG, Ahmed NM, Mohamed WS, Mabrouk MR (2020). Novel water-based coatings of acrylic polyurethane reinforced with mixed metal pigment for oil and gas pipelines protection. Progress Org. Coat..

[4] Li Z, Li Z (2023). EIS and Potentiodynamic polarization studies of arc-sprayed aluminum coating on Q235 steel surface. Int. J. Electrochem. Sci..

[5] Javidparvar A, Mosavi AM, Ramezanzadeh B (2023). Nickel-aluminium bronze (NiBRAl) casting alloy tribological/corrosion resistance properties improvement via deposition of a Cu-doped diamond-like carbon (DLC) thin film; optimization of sputtering magnetron process conditions. Mater. Chem. Phys..

[6] Amir F (2013). Electrochemical and structural properties of electroless Ni-P-SiC nanocomposite coatings. Appl. Surf Sci..

[7] Allahkaram SR (2011). Characterization and corrosion behavior of electroless Ni–P/nano-SiC coating inside the CO_2_ containing media in the presence of acetic acid. Mater. Design.

[8] Diebold, M., Backer, S. D., Niedenzu, P. M., Hester, B. R. & Vanhecke, F. A. Formulating paints with small particles. In *Pigments, Extenders, and Particles in Surface Coatings and Plastics: Fundamentals and Applications to Coatings, Plastics and Paper Laminate Formulation* (Springer, 2022).

[9] Ahmed NM, Mohamed MG, Abd-El-Gawad WM (2022). New out-performing structured pigments for anticorrosive practical applications. Pigment Resin Technol..

[10] Puig M, Gimeno MJ, Gracenea JJ, Suay JJ (2014). Anticorrosive properties enhancement in powder coating duplex systems by means of ZMP anticorrosive pigment. Assessment by electrochemical techniques. Progress Org. Coat..

[11] Arulvel S, Reddy DM, Rufuss DDW, Akinaga T (2021). A comprehensive review on mechanical and surface characteristics of composites reinforced with coated fibres. Surf. Interfaces.

[12] Pei LZ, Wei T, Lin N, Yu HY (2019). Synthesis of zinc oxide and titanium dioxide composite nanorods and their photocatalytic properties. Adv. Compos. Lett..

[13] Mohamed MG, Ahmed NM, Mohamed WS, Mabrouk MR (2020). Influence of anticorrosive coatings integrated with novel core-shell pigment on the corrosion protection of pipelines in CO2 environment. J. Mater. Eng. Perform..

[14] Zongxue Y (2015). Preparation of graphene oxide modified by titanium dioxide to enhance the anti-corrosion performance of epoxy coatings. Surf. Coat. Technol..

[15] Demian IN (2017). Understanding the anticorrosive protective mechanisms of modified epoxy coatings with improved barrier, active and self-healing functionalities: EIS and spectroscopic techniques. Sci. Rep..

[16] Joseph RX (2017). Application of EIS and SECM studies for investigation of anticorrosion properties of epoxy coatings containing zinc oxide nanoparticles on mild steel in 3.5% NaCl solution. J. Mater. Eng. Perform..

[17] Yu Z, Di H, Ma Y, He Y, Liang L, Lv L, Luo Z (2015). Preparation of graphene oxide modified by titanium dioxide to enhance the anti-corrosion performance of epoxy coatings. Surf. Coat. Technol..

[18] Morks MF, Fahim NF, Muster TH, Cole IS (2013). Cu-based Fe phosphate coating and its application in CO_2_ pipelines. Surf. Coat. Technol..

[19] Mousavifard SM, Nouri PM, Attar MM, Ramezanzadeh B (2013). The effects of zinc aluminum phosphate (ZPA) and zinc aluminum polyphosphate (ZAPP) mixtures on corrosion inhibition performance of epoxy/polyamide coating. J. Ind. Eng. Chem..

[20] Zubielewicz M, Langer E, Królikowska A, Komorowski L, Wanner M, Krawczyk K, Hilt M (2021). Concepts of steel protection by coatings with a reduced content of zinc pigments. Progress Org. Coat..

[21] Ahmed NM, Fathi AM, Mohamed MG, Abd-El-Gawad WM (2020). Evaluation of new core-shell pigments on the anticorrosive performance of coated reinforced concrete steel. Progress Org. Coat..

[22] Puig M, Cabedo L, Gracenea JJ, Jiménez-Morales A, Gámez-Pérez J, Suay JJ (2014). Adhesion enhancement of powder coatings on galvanised steel by addition of organo-modified silica particles. Progress Org. Coat..

